# Ultrasonography as a Diagnostic Support Tool for Childhood Takayasu Arteritis Referred to as Fever of Unknown Origin: Case Series and Literature Review

**DOI:** 10.31662/jmaj.2020-0115

**Published:** 2021-09-13

**Authors:** Hisataka Nozawa, Masao Ogura, Mikiko Miyasaka, Hiromichi Suzuki, Kenji Ishikura, Akira Ishiguro, Shuichi Ito

**Affiliations:** 1 Center for Postgraduate Education and Training, National Center for Child Health and Development, Tokyo, Japan; 2 Division of Pediatric Nephrology and Rheumatology, National Center for Child Health and Development, Tokyo, Japan; 3 Department of Radiology, National Center for Child Health and Development, Tokyo, Japan; 4 Department of Health Policy, National Research Institute for Child Health and Development, Tokyo, Japan; 5 Department of Pediatrics, Graduate School of Medicine, Yokohama City University, Yokohama, Japan

**Keywords:** Takayasu arteritis, pediatrics, diagnostic imaging, fever of unknown origin, ultrasonography

## Abstract

**Introduction:**

Childhood Takayasu arteritis (c-TA) often shows nonspecific symptoms, such as fever of unknown origin (FUO). Delay of diagnosis may result in organ dysfunction by arterial occlusion; therefore, early diagnosis is very important. Although ultrasonography is the first-line screening tool for children with FUO, its diagnostic efficacy of evaluation of systemic arteries in c-TA that presents as FUO remains unclear. We evaluated the suitability of ultrasonography evaluation that included systemic vessels for the early diagnosis of c-TA initially presenting as FUO.

**Methods:**

We review five patients who received a diagnosis of c-TA in our institution and also performed a literature review regarding TA cases with FUO and diagnosed on the basis of initial ultrasonography.

**Results:**

As in our cases, the median period from onset to diagnosis was 25 days (interquartile range [IQR], 21-35). Comparing the initial ultrasonography findings with later contrast-enhanced computed tomography (CECT) findings in the abdominal aorta, celiac artery, superior mesenteric artery, bilateral renal arteries, and bilateral common carotid arteries, the concordance rate between ultrasonography and CECT was moderate (Kappa coefficient was 0.50). All the patients were successfully treated without severe vascular damage. The literature review revealed 12 articles; although 9 of the 13 patients did not show the characteristic features (such as blood pressure discrepancy, bruit, or pulse deficiency), the median time to diagnosis was still 5 months (IQR, 3-12).

**Conclusions:**

During initial screening for patients with FUO, ultrasonography including evaluation of systemic vessels could contribute to earlier diagnosis of c-TA.

## Introduction

Takayasu arteritis (TA) is a large vessel vasculitis affecting the aorta and its major branches. Childhood TA (c-TA) is a very rare disease, with an annual incidence in the pediatric population of approximately 2/1,000,000 ^(1)^. Additionally, during the early stages, its typical clinical manifestations (pulseless upper extremities, leg claudication, and fainting) are often lacking. Furthermore, c-TA often presents as a fever of unknown origin (FUO). Kasai et al. reported that 4.8% of children with FUO eventually received a diagnosis of c-TA ^(2)^. Additionally, approximately 80% of patients with c-TA actually demonstrated fever ^(3)^, although that is a relatively minor symptom in adult TA (<34.7%) ^(4)^. Early diagnosis of c-TA is challenging for pediatricians but is very important because delay in diagnosis and intervention may result in vascular stenosis, dilation, and obstruction, which are often followed by organ dysfunction.

The appropriate criteria from the American College of Radiology state that ultrasonography may be appropriate for the imaging modality evaluating pediatric patients with FUO ^(5)^ because it is free from radiation exposure and the need for sedation. The recommendations from the European League Against Rheumatism (EULAR) mention the early diagnosis by ultrasonography ^(6)^. However, the systematic review of the diagnosis of large vessel vasculitis states that there was no high-quality literature evaluating the usefulness of ultrasonography in the diagnosis of TA ^(7)^. If we could detect abnormal imaging findings of TA among patients with FUO with ultrasonography screening, we would immediately select essential radiological diagnostic procedures for definitive diagnosis of TA, such as contrast-enhanced computed tomography (CECT). To the best of our knowledge, no literature has reviewed the contribution of ultrasonography screening to the diagnosis of c-TA in patients with FUO. On the basis of the experience of pediatric cases diagnosed as c-TA in patients with FUO, we evaluated using case literature review the suitability of ultrasonography evaluation that included systemic vessels for the early diagnosis of c-TA initially presenting as FUO.

## Materials and Methods

This study was performed in line with the principles of the Declaration of Helsinki. Approval was granted by the Ethics Committee of the National Center for Child Health and Development on July 27, 2017 (number 1493), and written informed consent was obtained from all patients.

### Patients and measurements

We included all patients who were referred as FUO to the National Center for Child Health and Development in Tokyo between March 2002 and March 2016 and who finally received a diagnosis of c-TA. Definitive diagnosis was based on EULAR, Paediatric Rheumatology International Trials Organisation (PRINTO), and Paediatric Rheumatology European Society (PRES) criteria for TA ^(8)^. We excluded patients who already had suspected c-TA by several radiological procedures performed in previous hospitals and were referred for treatment of c-TA. We investigated their clinical findings, including sex, age of onset, laboratory examinations (C-reactive protein level and erythrocyte sedimentation rate), duration from onset to diagnosis, Indian Takayasu Activity Score 2010 (ITAS2010) at diagnosis and at the latest ultrasonography follow-up date until July 2020, treatment, and Vasculitis Damage Index (VDI) at the latest ultrasonography follow-up date until July 2020.

We also compared the ultrasonography and CECT findings of our patients regarding the following seven vessels: abdominal aorta (AAo), celiac artery, superior mesenteric artery (SMA), right and left renal arteries, and right and left common carotid arteries (CCAs).

### Search strategy and study selection

We searched PubMed, Ovid/MEDLINE, Embase, and Cochrane library databases for previously published studies until August 31, 2020. In each database, the search query was the following: (“Takayasu arteritis”[MeSH Terms] OR “takayasu*”[Title/Abstract]) AND (“ultrasonography”[MeSH Terms] OR (“ultrasound”[Title/Abstract] OR “ultrasonography”[Title/Abstract] OR “sonography”[Title/Abstract])) in PubMed and Ovid/MEDLINE and (‘aortic arch syndrome’/exp OR ‘takayasu*’:ab,ti) AND (‘echography’/exp OR ‘ultrasound’:ab,ti OR ‘ultrasonography’:ab,ti OR ‘sonography’:ab,ti) AND [english]/lim AND [embase]/lim in Embase, ([Takayasu Arteritis] explode all trees) AND ([Ultrasonography] explode all trees) in Cochrane library. English language filter was activated in PubMed and Ovid/MEDLINE. We also performed a manual search of reference literature from the full text available studies.

Case reports about the diagnosis of TA by the initial ultrasonography for patients with FUO were included. Review articles and the literature lacking the correct clinical course for individual patients were excluded.

## Results

### Characteristics and clinical manifestations of enrolled patients

During this study period, five patients received a diagnosis of c-TA. Table 1 shows the summary of the five patients. All patients had suspected TA by the initial screening ultrasonography and then received a definitive diagnosis on the follow-up CECT. They fulfilled the EULAR-PRINTO-PRES diagnostic criteria. The median age at onset was 11 years (interquartile range [IQR], 9-12 years). The median duration from onset to diagnosis was 25 days (IQR, 21-35 days). The median value of ITAS2010 at diagnosis was 3 (IQR, 1-9). All patients received immunosuppressive treatment, and the value of ITAS2010 decreased to 0 at the most recent ultrasonography follow-up date. The highest value of VDI among five patients was 2.

**Table 1.** Summary of Clinical Manifestations of Five Patients.

Case No.	Sex/Age at onset	Clinical symptoms other than fever	Applicable number to EULAR-PRINTO-PRES criteria/presence of bruit	Laboratory data at diagnosis		Duration from onset to diagnosis (days)	ITAS2010 at diagnosis	Treatment		Follow-up status at most recent US date		
				CRP (mg/dL)	ESR (mm/h)			Induction therapy	Present therapy	ITAS2010	VDI	Follow-up period (months)
1	F/12 years	Knee pain	3/Yes	7.8	129	25	9	MPT	PSL/AZP/MTX	0	1	53
2	M/9 years	Abdominal pain	1/No	8.2	132	21	1	MPT	PSL/AZP	0	1	55
3	M/12 years	Abdominal pain	2/Yes	10.4	106	15	3	MPT/IVCY	MMF	0	2	70
4	F/11 years	Lymphadenopathy	4/Yes	3.0	113	35	11	MPT/IVCY	None	0	1	91
5	F/5 months	Cough	2/No	11.5	132	37	0	MPT/IVCY	None	0	0	60

AZP, azathioprine; CRP, C-reactive protein; ESR, erythrocyte sedimentation rate; EULAR, European League Against Rheumatism; F, female; IVCY, intravenous cyclophosphamide therapy; ITAS, Indian Takayasu activity score, M, male; MMF, mycophenolate mofetil; MPT, methylprednisolone pulse therapy; MTX, methotrexate; PRES, Pediatric Rheumatology European Society; PRINTO, Pediatric Rheumatology International Trials Organization; PSL, prednisolone; VDI, vasculitis damage index

### Ultrasonography and CECT findings

**Figure 1.**
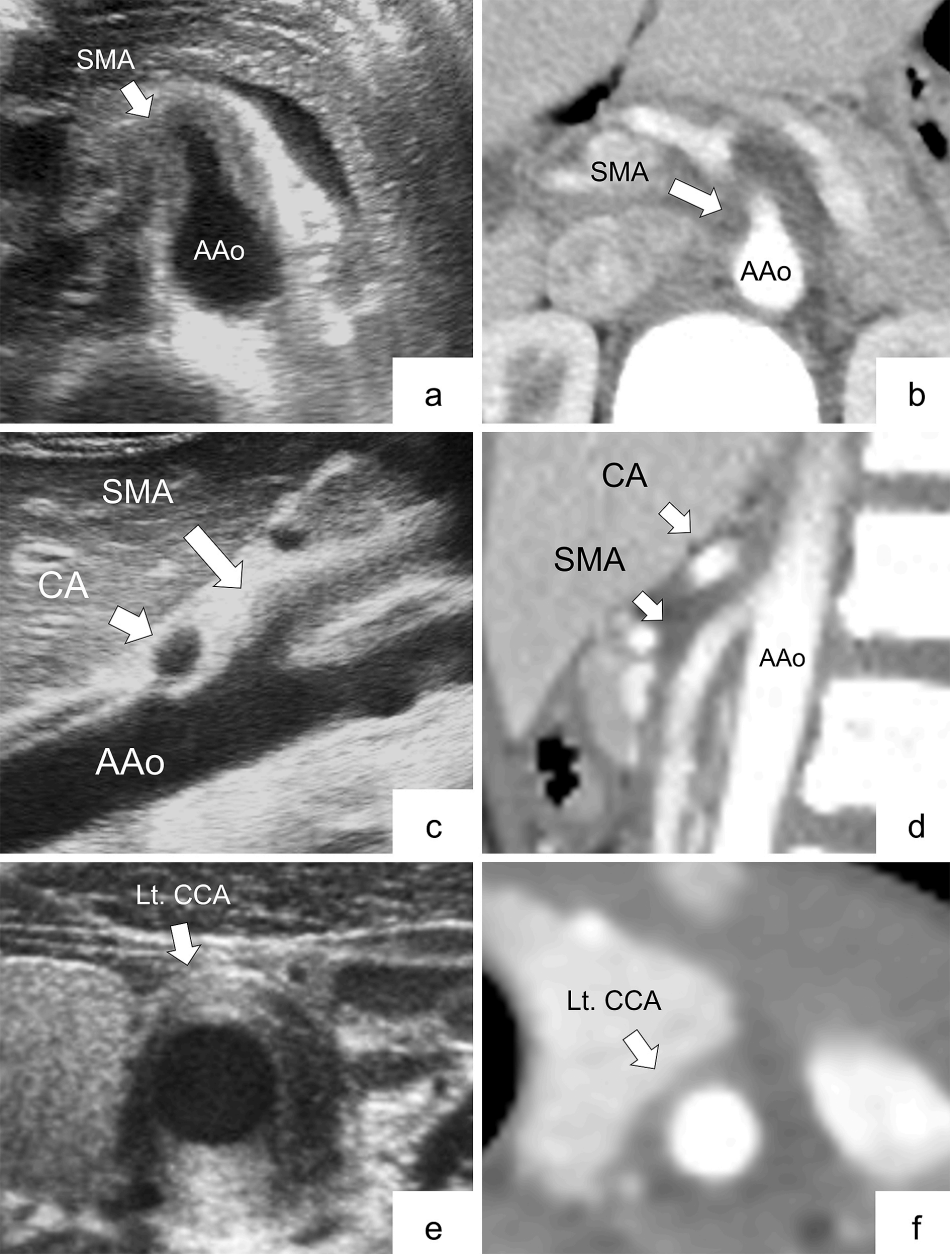
Representative images of arterial involvement comparing ultrasonography with contrast-enhanced computed tomography (CECT) in Patient No. 3 (panels a, b, c, d, e, and f). a: Transverse view shows wall thickening at the level of the trunk of the superior mesenteric artery (SMA) in the ultrasonogram (arrow). b: CECT at the same level of the abdominal ultrasonogram demonstrates wall thickening of the SMA (arrow). c: Longitudinal view shows arterial stenosis at the level of the trunk of the celiac artery (CA), and wall thickening of the SMA in the ultrasonogram (arrow). d: CECT at the same level of the abdominal ultrasonogram recognizes both findings of the CA and SMA (arrow). e: Wall thickening of the left common carotid artery (CCA) is shown in the transverse view in the ultrasonogram (arrow). f: Wall thickening of the left CCA is revealed by CECT at the same level of the neck ultrasonogram (arrow). AAo, abdominal aorta; CA, celiac artery; CCA, common carotid artery; Lt., left; SMA, superior mesenteric artery.

Table 2 shows the radiological findings of ultrasonography and CECT for seven arteries. Figure 1 (panels a, b, c, d, e, and f) shows the representative ultrasonograms. All findings were classified as follows: 9 arteries were positive on both ultrasonography and CECT (6 abdominal and 3 cervical), 2 were positive on ultrasonography alone (1 abdominal and 1 cervical), 6 were positive on CECT alone (4 abdominal and 2 cervical), and 16 were negative on both ultrasonography and CECT (14 abdominal and 2 cervical). For patient 5, bilateral CCAs were not evaluated by either ultrasonography or CECT. There was moderate agreement between ultrasonography and CECT assessment on all arteries and abdominal arteries (Kappa coefficients were 0.50 and 0.56).

**Table 2.** Arterial Findings Compared between Ultrasonography and Contrast-enhanced Computed Tomography.

Case No.	Abdominal arteries (US/CECT)					Cervical arteries (US/CECT)		US undetectable lesions detected by CECT
	AAo	CA	Right RA	Left RA	SMA	Right CCA	Left CCA	
1	−/＋	−/−	−/＋	−/−	＋/＋	−/−	＋/＋	Wall thickening of the left subclavian artery and trunk of right renal artery
2	−/－	−/−	−/−	−/−	−/−	−/＋	＋/＋	None
3	−/−	＋/＋	−/−	−/−	＋/＋	−/−	＋/＋	None
4	−/＋	−/＋	−/−	−/−	＋/＋	＋/−	−/＋	None
5	＋/＋	＋/＋	−/−	−/−	＋/−	NA	NA	None

AAo, abdominal aorta; CA, celiac artery; CCA, common carotid artery; CECT, contrast-enhanced computed tomography; NA, not available; RA, renal artery; SMA, superior mesenteric artery; US, ultrasonography

### Study review

**Figure 2.**
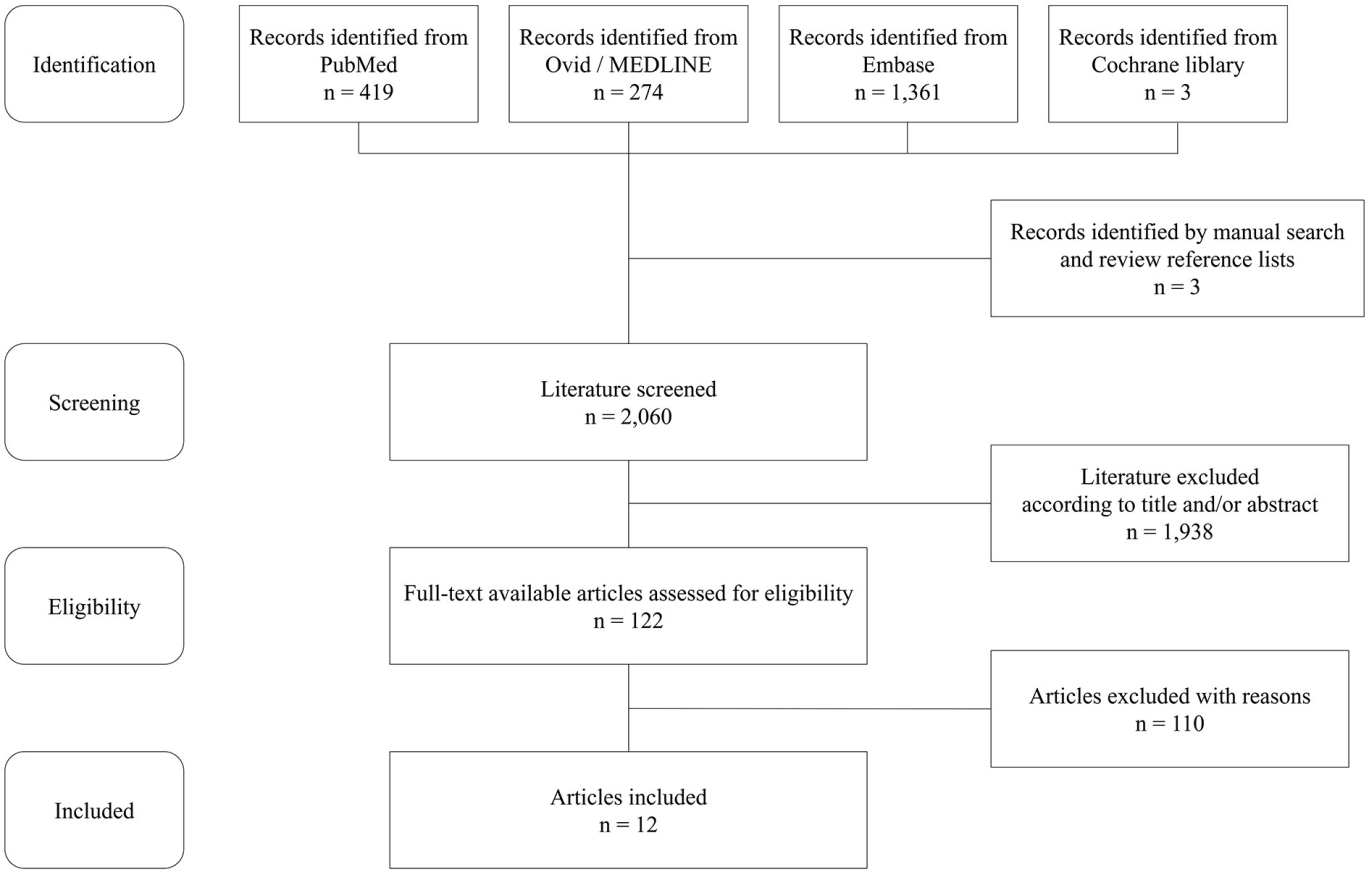
Flowchart of the literature review on Takayasu arteritis diagnosed with ultrasonography among patients with fever of unknown origin.

According to the search strategy, inclusion and exclusion criteria, 12 articles including 13 cases were reviewed ^(9), (10), (11), (12), (13), (14), (15), (16), (17), (18), (19), (20)^. Figure 2 shows a flowchart depicting the article screening and selection. Table 3 shows the summary of all patients in the reviewed articles. Only three of 13 patients showed specific manifestations, including bruit, pulse deficit, or blood pressure discrepancy. The median duration from onset to diagnosis was 5 months (IQR, 3-12 months).

**Table 3.** Summary of Literature Review on TA Cases Detected by Initial US Screening among Patients with Fever of Unknown Origin.

Study	Sex/Age at onset (years)	Clinical symptoms other than fever	Applicable number to EULAR-PRINTO-PRES criteria/Presence of a bruit	Laboratory data at diagnosis		Duration from onset to diagnosis (months)	US/Definitive image		Modality for definitive diagnosis	Follow-up period (months)	Present therapy	Relapse
				CRP (mg/dL)	ESR (mm/h)		Abdominal	Cervical/Thoracic				
Tachibana et al	F/20	Pain throughout entire body	1/No	7.5	99	4	NA/+	+/+	CTA	6	PSL/MTX	Yes
Goel et al	M/18	Dyspnea	1/No	6.0	35	6	+/+	+/+	CTA	3	PSL/MTX	No
Sasae et al	F/20	Periumbilical pain	2/No	9.1	119	3	+/+	+/+	CTA	4	PSL	No
Sada et al	F/19	Malaise	1/No	31.2	145	6	NA/NA	+/+	CTA	24	PSL/MTX	No
Agostinis et al	M/75	Cough and neck pain	1/No	15.1	85	3	NA/+	+/+	FDG-PET	6	PSL/MTX	No
Gupta et al	F/20	Painful nodular skin lesions	2/No	15.2	77	12	+/+	NA/+	MRA	NA	PSL	No
Ahasan et al	F/20	Pain in all four limbs	2/No	1.2	62	2	+/NA	NA/NA	NA	NA	PSL	NA
Thai et al	F/14	Pain in left upper extremity	4/Yes	21000.0	154	4	NA/NA	+/NA	MRA	NA	PSL	NA
Skoura et al	F/54	None	1/No	2.0	80	NA	+/NA	NA/+	FDG-PET	6	PSL	NA
Uthman et al	F/28	None	1/No	NA	150	18	NA/NA	+/+	DSA	5	PSL/MTX	NA
KiŞla Ekİncİ et al	F/12	Painful lesion on the face	1/No	14.8	116	2	+/+	+/+	MRA	18	PSL/MTX/MM F	Yes
Schmidt et al	F/38	Joint pain	1/No	3.6	92	12	NA/NA	+/+	DSA	14	PSL/AZP	No
	F/27	Joint pain	1/No	24.6	120	36	NA/NA	+/+	DSA	8	PSL/MTX	Yes

AZP, azathioprine; CTA, computed tomography angiography; CRP, C-reactive protein; DSA, digital subtraction angiography; ESR, erythrocyte sedimentation rate; EULAR, European League Against Rheumatism; F, female; FDG-PET, [18F]-fluorodeoxyglucose positron emission tomography; M, male; MMF, mycophenolate mofetil; MRA, magnetic resonance angiography; MTX, methotrexate; NA, not available; PRES, Pediatric Rheumatology European Society; PRINTO, Pediatric Rheumatology International Trials Organization; PSL, prednisolone; US, ultrasonography

## Discussion

According to the present study, it is important for early diagnosis of c-TA to perform ultrasonography screening that includes large systemic vessels (as many as we can observe) among children with FUO. Detection of ultrasonography abnormalities may lead to confirmative examinations, such as via CECT, and early diagnosis of c-TA contributed to favorable patient outcomes. The diagnosis of TA is generally made by a combination of imaging modalities. Our study showed that ultrasonography was able to detect abnormal arteries in the abdomen and neck regions with a moderate concordance rate compared with CECT. In the early stage of TA, patients show only nonspecific symptoms, such as persistent fever. Additionally, in the so-called “pre-pulseless phase” of vascular stenosis, pulse deficit or blood pressure discrepancy, which are hallmarks of TA, may be lacking ^(21)^. However, both in our patients and in cases from the review articles, the median duration from onset to definitive diagnosis tends to be shorter than those stated in the previous studies ^(22), (23), (24), (25)^. Furthermore, ultrasonography is minimally invasive (free from radiation exposure or sedation) and low in cost and provides satisfactory access to images. Thus, it is preferable as a screening and diagnostic support tool for c-TA among children with FUO.

There are two reasons why ultrasonography evaluation of systemic arteries, particularly including those in the abdomen, is suitable for c-TA patients among children with FUO: first, different distribution of arterial involvement in c-TA compared with that in adult TA and, second, favorable body habitus of children for ultrasonography screening.

Recent reports revealed that c-TA is more likely to demonstrate subphrenic aorta involvement rather than in intrathoracic or suprathoracic regions ^(26), (27), (28)^. Our results were also consistent with this presentation. A retrospective cohort study of c-TA in the United States reported that the frequency of abdominal involvement was 52.4% in the AAo and 42.9% in the SMA, and (in the thorax and neck) 38.1% in the right CCA and 42.9% in the left CCA ^(29)^. Feng et al. reported arterial involvement in 11 patients with c-TA. All 11 patients had involvement of the AAo and its branches, but only three (27.2%) had involvement of the CCAs ^(30)^.

As mentioned above, the body habitus of children is favorable for abdominal ultrasonography screening compared with that of adults. They have a thinner abdominal wall and less fat compared with adults ^(31)^, thus enabling higher-resolution ultrasonography images. The “macaroni sign,” which is a long, smooth, homogeneous, and moderately echogenic circumferential thickening of the cervical arteries on the transverse section, specific for early TA ^(32), (33), (34)^, is similarly observed in the abdominal region in our patients. In adults, abdominal arteries are often obscured because ultrasonography cannot visualize them because of a larger physique with thick subcutaneous tissue or large volumes of bowel gas. Thus, in adult TA, the efficacy of ultrasonography is mainly limited to CCAs ^(35), (36), (37)^. Among the cases in the review articles, the patients’ median age whose involvement of the abdominal arteries could be detected by ultrasonography was 20 years (range, 12-54 years), which tended to be young.

**Figure 3.**
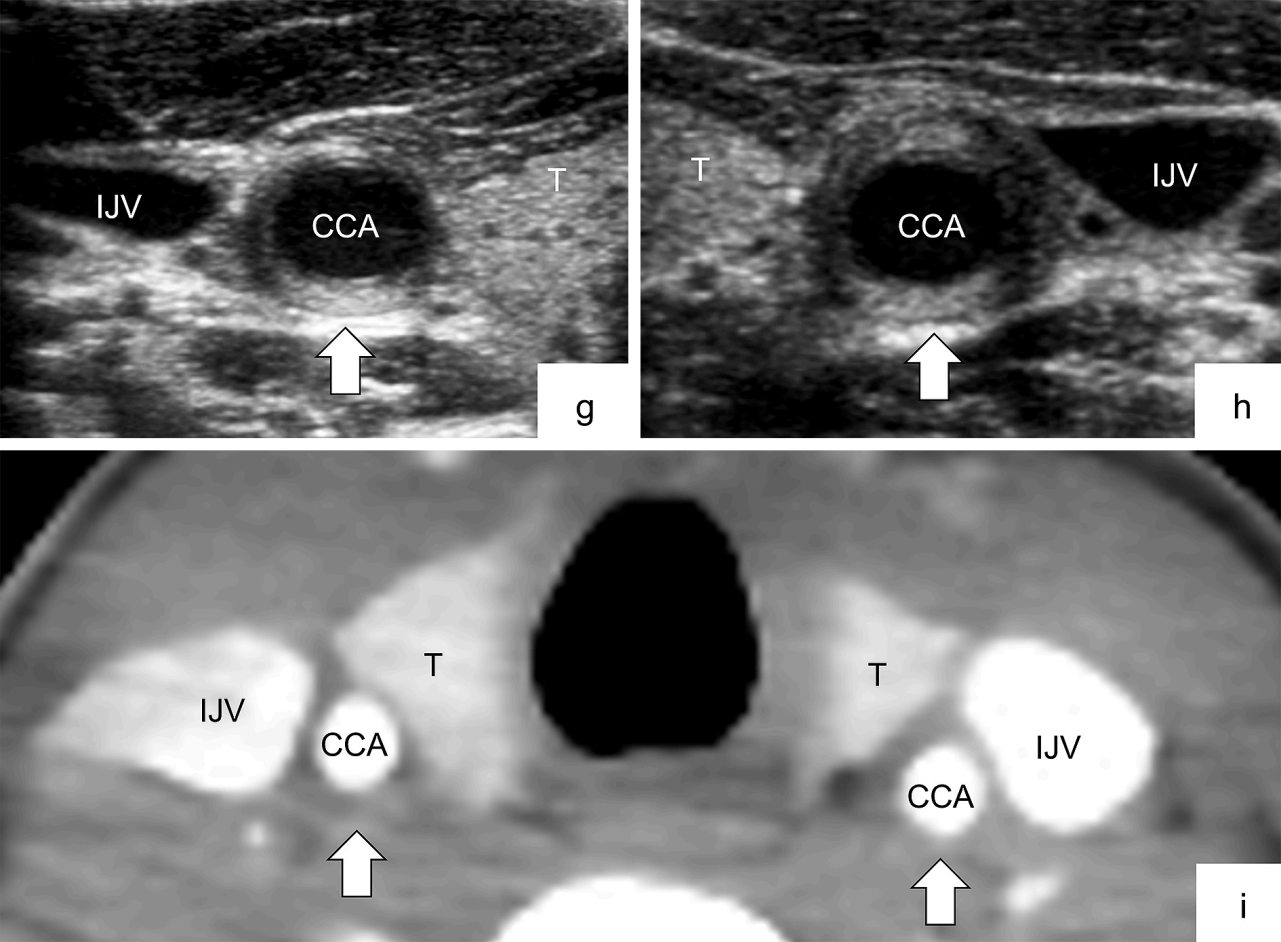
Transverse imaging of bilateral CCAs in Patient No. 4 (panels g to i). g and h: Thickening at the dorsal walls of bilateral CCAs is clearly detected via ultrasonography (panel g shows the right side, and panel h shows the left side). i: Wall thickening at the same level is unclear in CECT. CCA, common carotid artery; IJV, internal jugular vein; T, thyroid gland.

Ultrasonography findings did not completely correspond with CECT findings in the cervical regions. The Kappa coefficient, which indicates the concordance rate between ultrasonography and CECT, also tended to be slightly higher in the abdominal region only than in the abdominal and cervical regions together. It may suggest that cervical CECT may be inferior to abdominal CECT in diagnostic accuracy. As subcutaneous fat and tissue during childhood are significantly thinner in the neck area than in adults, it is difficult to distinguish the arterial wall from the periarterial soft tissue in CECT. Additionally, technical problems, such as motion artifact or inaccurate scan setting (timing and slice thickness), can affect image quality. These also apply to magnetic resonance imaging (MRI) and may be a common caveat particularly in children who are small and difficult to immobilize for a long time. Furthermore, if the arterial abnormality (i.e., wall thickening) is localized in a small section of the carotid artery, as shown in Figure 3 (panels g, h, and i), CECT may not differentiate such small vascular abnormalities. As CECT or MRI is not always a definitive imaging modality, additional ultrasonography examination should be considered when c-TA is suspected.

Although the results of our study encourage initial ultrasonography screening of systemic arteries to find c-TA in children with FUO, our study has several limitations. First, this study was a retrospective analysis with small numbers of patients at a single center. Second, TA is a more well-known and prevalent disease among Japanese and other Asian populations than among those of Europe and the United States. Third, ultrasonography is dependent on observers’ skills and cannot visualize the proximal subclavian artery and thoracic aorta because of the interference of the costal bones and the normal presence of air in the lung. Finally, we compared only ultrasonography and CECT but did not include contrast-enhanced ultrasonography (CEUS) or MRI. Recently, CEUS and MRI have been added as effective tools for the diagnosis and assessment of disease activity in TA ^(32), (37), (38), (39)^. CEUS has not yet been introduced in our institution. When screening for FUO, we performed CECT as a secondary survey prior to MRI to search for fever sources other than arteritis. Considering that some patients at the susceptible age for TA do not always require sedation during short-term MRI, in the future, we intend to perform MRI focusing on abnormal arteries to confirm the diagnosis of TA.

### Conclusion

Ultrasonography is preferable as a screening and diagnostic support tool for c-TA referred to as pediatric patients with FUO. For early diagnosis of c-TA, it is important to perform ultrasonography screening that includes large systemic vessels (as many as we can observe) among children with FUO.

## Article Information

### Conflicts of Interest

Shuichi Ito has the following conflicts of interest relevant to this article to disclose: an advisory fee from Takeda Pharmaceutical, and the lecture fees from Chugai Pharmaceutical, Mitsubishi Tanabe Pharma, Pfizer, and Takeda Pharmaceutical, and research funding from Chugai Pharmaceutical and Mitsubishi Tanabe Pharma.

Kenji Ishikura has the following conflicts of interest relevant to this article to disclose: honoraria from Asahi Kasei Pharma, Chugai Pharmaceutical, Novartis Pharma, and Zenyaku Kogyo, and research funding from Chugai Pharmaceutical and Zenyaku Kogyo.

### Acknowledgement

We thank the medical editor from the Division of Education for Clinical Research, National Center for Child Health and Development for editing this article.

### Author Contributions

All authors contributed to the conception and design of the study. Hisataka Nozawa provided material preparation, data collection, interpretation, and participated in the design, and revision of the review and wrote the first draft of the manuscript. Mikiko Miyasaka revised figures and figure legends. Hiromichi Suzuki developed the search strategy for searches. Shuichi Ito supervised the entire process. All authors commented on previous versions of the manuscript, approved the final manuscript as submitted, and agreed to be accountable for all aspects of the work.

### Disclaimer

Shuichi Ito is one of the Editors of JMA Journal and on the journal’s Editorial Staff. He was not involved in the editorial evaluation or decision to accept this article for publication at all.

### Approval by Institutional Review Board (IRB)

This study was performed in line with the principles of the Declaration of Helsinki. Informed consent was obtained from all individual participants included in this study and their parents. The participants and their parents consented to the submission of the case series to the journal. Approval was granted by the Ethics Committee of the National Center for Child Health and Development on July 27, 2017 (number 1493).

## References

[1] Russo RAG, Katsicas MM. Takayasu arteritis. Front Pediatr. 2018;6:265. 30338248 10.3389/fped.2018.00265 PMC6165863

[2] Kasai K, Mori M, Hara R, et al. National survey of childhood febrile illness cases with fever of unknown origin in Japan. Pediatr Int. 2011;53(4):421-5. 21105965 10.1111/j.1442-200X.2010.03296.x

[3] Maeda M, Kobayashi M, Okamoto S, et al. Aortitis syndrome in children: clinical observation of 35 cases in Japan. Acta Paediatr Jpn. 1997;39(2):280-4. 9141273 10.1111/j.1442-200x.1997.tb03600.x

[4] Watanabe Y, Miyata T, Tanemoto K. Current clinical features of new patients with Takayasu arteritis observed from cross-country research in Japan: age and sex specificity. Circulation. 2015;132(18):1701-9. 26354799 10.1161/CIRCULATIONAHA.114.012547

[5] Westra SJ, Karmazyn BK, Alazraki AL, et al. ACR appropriateness criteria fever without source or unknown origin―child. J Am Coll Radiol. 2016;13(8):922-30. 27374781 10.1016/j.jacr.2016.04.028

[6] Bardi M, Diamantopoulos AP. EULAR recommendations for the use of imaging in large vessel vasculitis in clinical practice summary. Radiol Med. 2019;124(10):965-72. 31254221 10.1007/s11547-019-01058-0

[7] Duftner C, Dejaco C, Sepriano A, et al. Imaging in diagnosis, outcome prediction and monitoring of large vessel vasculitis: a systematic literature review and meta-analysis informing the EULAR recommendations. RMD Open. 2018;4(1):e000612. 29531788 10.1136/rmdopen-2017-000612 PMC5845406

[8] Ozen S, Pistorio A, Iusan SM, et al. EULAR/PRINTO/PRES criteria for Henoch-Schonlein purpura, childhood polyarteritis nodosa, childhood Wegener granulomatosis and childhood Takayasu arteritis: Ankara 2008. Part II: Final classification criteria. Ann Rheum Dis. 2010;69(5):798-806. 20413568 10.1136/ard.2009.116657

[9] Tachibana M, Mukouhara N, Hirami R, et al. Ultrasonography is convenient and useful for assessment and follow-up of Takayasu’s arteritis. J Med Ultrason (2001). 2014;41(3):365-9. 27277912 10.1007/s10396-013-0515-7

[10] Goel PK, Moorthy N, Kumar S. The role of noninvasive imaging in early diagnosis of clinically masked prepulseless inflammatory phase of Takayasu’s arteritis. Echocardiography. 2012;29(1):59-63. 22098565 10.1111/j.1540-8175.2011.01581.x

[11] Sasae Y, Morita Y, Sakuta T, et al. Abdominal pain as the initial presentation of Takayasu arteritis. Mod Rheumatol. 2008;18(5):496-8. 18463944 10.1007/s10165-008-0075-7

[12] Sada E, Kohno M, Iwamasa K, et al. Clinical usefulness of multiplanar reconstruction images obtained by multi-slice computed tomographic angiography for early-stage Takayasu’s arteritis. Mod Rheumatol. 2004;14(3):245-9. 17143683 10.1007/s10165-004-0300-y

[13] Agostinis P, Antonello RM, Orsaria M, et al. Isoniazid-induced Takayasu arteritis remission. Infez Med. 2019;27(4):436-40. 31846995

[14] Gupta M, Singh K, Lehl SS, et al. Recurrent erythema nodosum: a red flag sign of hidden systemic vasculitis. BMJ Case Rep. 2013;2013:bcr2013009507. 10.1136/bcr-2013-009507 PMC3645399 23576669

[15] Ahasan HAMN, Alam B, Chowdhury MH, et al. Takayasu’s arteritis in association with tuberculosis in a young woman. Pak J Med Sci. 2009;25(6):1009-11.

[16] Tsai MJ, Lin SC, Wang JK, et al. A patient with familial Takayasu’s arteritis presenting with fever of unknown origin. J Formos Med Assoc. 1998;97(5):351-3. 9610060

[17] Skoura E, Giannopoulou C, Keramida G, et al. A case of fever of unknown origin: (18)F-FDG-PET/CT findings in Takayasu’s arteritis. Hell J Nucl Med. 2008;11(3):172-4. 19081862

[18] Uthman IW, Bizri AR, Hajj Ali RA, et al. Takayasu’s arteritis presenting as fever of unknown origin: report of two cases and literature review. Semin Arthritis Rheum. 1999;28(4):280-5. 10073502 10.1016/s0049-0172(99)80023-7

[19] KiŞla Ekİncİ RM, Balci S, PİŞkİn FC, et al. Pre-pulseless Takayasu arteritis in a child represented with prolonged fever of unknown origin and successful management with concomitant mycophenolate mofetil and infliximab. Arch Rheumatol. 2020;35(2):278-82. 32851379 10.46497/ArchRheumatol.2020.7599 PMC7406164

[20] Schmidt W, Nerenheim A, Seipelt E, et al. Diagnosis of early Takayasu arteritis with sonography. Rheumatology (Oxford). 2002;41(5):496-502. 12011371 10.1093/rheumatology/41.5.496

[21] Yotsuyanagi H, Chikatsu N, Kaneko Y, et al. Takayasu’s arteritis in prepulseless stage manifesting lymph node swelling and hepatosplenomegaly. Intern Med. 1995;34(5):455-9. 7647421 10.2169/internalmedicine.34.455

[22] Kerr GS, Hallahan CW, Giordano J, et al. Takayasu arteritis. Ann Intern Med. 1994;120(11):919-29. 7909656 10.7326/0003-4819-120-11-199406010-00004

[23] Sahin S, Hopurcuoglu D, Bektas S, et al. Childhood-onset Takayasu arteritis: a 15-year experience from a tertiary referral center. Int J Rheum Dis. 2019;22(1):132-9. 30397997 10.1111/1756-185X.13425

[24] Aeschlimann FA, Barra L, Alsolaimani R, et al. Presentation and disease course of childhood-onset versus adult-onset Takayasu arteritis. Arthritis Rheumatol. 2019;71(2):315-23. 30101446 10.1002/art.40690

[25] Clemente G, Silva CA, Sacchetti SB, et al. Takayasu arteritis in childhood: misdiagnoses at disease onset and associated diseases. Rheumatol Int. 2018;38(6):1089-94. 29687155 10.1007/s00296-018-4030-4

[26] Clemente G, Hilario MO, Len C, et al. Brazilian multicenter study of 71 patients with juvenile-onset Takayasu’s arteritis: clinical and angiographic features. Rev Bras Reumatol Engl Ed. 2016;56(2):145-51. 27267528 10.1016/j.rbre.2016.01.004

[27] Mathew AJ, Goel R, Kumar S, et al. Childhood-onset Takayasu arteritis: an update. Int J Rheum Dis. 2016;19(2):116-26. 26585174 10.1111/1756-185X.12718

[28] Goel R, Kumar TS, Danda D, et al. Childhood-onset Takayasu arteritis―experience from a tertiary care center in South India. J Rheumatol. 2014;41(6):1183-9. 24786922 10.3899/jrheum.131117

[29] Szugye HS, Zeft AS, Spalding SJ. Takayasu arteritis in the pediatric population: a contemporary United States-based single center cohort. Pediatr Rheumatol Online J. 2014;12:21. 24955077 10.1186/1546-0096-12-21 PMC4065084

[30] Feng Y, Tang X, Liu M, et al. Clinical study of children with Takayasu arteritis: a retrospective study from a single center in China. Pediatr Rheumatol Online J. 2017;15(1):29. 28416004 10.1186/s12969-017-0164-2 PMC5393038

[31] Siegel MJ. Pediatric sonography. 4th ed. Philadelphia: Wolters Kluwer/Lippincott Williams & Wilkins; 2011. 725 p.

[32] Schmidt WA. Role of ultrasound in the understanding and management of vasculitis. Ther Adv Musculoskelet Dis. 2014;6(2):39-47. 24688604 10.1177/1759720X13512256 PMC3956137

[33] Chaubal N, Dighe M, Shah M. Sonographic and color Doppler findings in aortoarteritis (Takayasu arteritis). J Ultrasound Med. 2004;23(7):937-44. 15292562 10.7863/jum.2004.23.7.937

[34] Matsunaga N, Hayashi K, Sakamoto I, et al. Takayasu arteritis: protean radiologic manifestations and diagnosis. Radiographics. 1997;17(3):579-94. 9153698 10.1148/radiographics.17.3.9153698

[35] Kissin EY, Merkel PA. Diagnostic imaging in Takayasu arteritis. Curr Opin Rheumatol. 2004;16(1):31-7. 14673386 10.1097/00002281-200401000-00007

[36] Pipitone N, Versari A, Salvarani C. Role of imaging studies in the diagnosis and follow-up of large-vessel vasculitis: an update. Rheumatology (Oxford). 2008;47(4):403-8. 18292120 10.1093/rheumatology/kem379

[37] Gotway MB, Araoz PA, Macedo TA, et al. Imaging findings in Takayasu’s arteritis. AJR Am J Roentgenol. 2005;184(6):1945-50. 15908559 10.2214/ajr.184.6.01841945

[38] Czihal M, Lottspeich C, Hoffmann U. Ultrasound imaging in the diagnosis of large vessel vasculitis. Vasa. 2017;46(4):241-53. 28332442 10.1024/0301-1526/a000625

[39] Soliman M, Laxer R, Manson D, et al. Imaging of systemic vasculitis in childhood. Pediatr Radiol. 2015;45(8):1110-25. 26198677 10.1007/s00247-015-3339-3

